# Low Concentrations of Corticosterone Exert Stimulatory Effects on Macrophage Function in a Manner Dependent on Glucocorticoid Receptors

**DOI:** 10.1155/2013/405127

**Published:** 2013-10-01

**Authors:** He-Jiang Zhong, Hai-Yan Wang, Ce Yang, Jian-Yun Zhou, Jian-Xin Jiang

**Affiliations:** ^1^Department of Anesthesiology, Xinqiao Hospital, Third Military Medical University, Chongqing 400037, China; ^2^State Key Laboratory of Trauma, Burns, and Combined Injury, Research Institute of Surgery, Daping Hospital, Third Military Medical University, Chongqing 400042, China; ^3^Medical Department, Xinqiao Hospital, Third Military Medical University, Chongqing 400037, China

## Abstract

Endogenous glucocorticoids (GCs) have both stimulatory and suppressive effects on immune cells depending on the concentration. However, the mechanisms underlying the stimulatory effects of GCs remain elusive. Rat peritoneal macrophages were treated with different concentrations of corticosterone (0, 30 nM, 150 nM, and 3 **μ**M). To inhibit the glucocorticoid receptor (GR) activity, macrophages were preincubated with the GR antagonist RU486 (mifepristone, 10 **μ**M) for 30 min before treatment with corticosterone (150 nM). In the absence of immune stimuli, the chemotactic and phagocytic activities of macrophages were markedly enhanced by low concentrations of corticosterone (30 and 150 nM) when compared with vehicle-treated controls. However, these effects were not observed at a high concentration of corticosterone (3 **μ**M). Furthermore, blocking GR activity inhibited 150 nM corticosterone-enhanced chemotaxis and phagocytosis of macrophages. Meanwhile, after treatment with corticosterone (150 nM) for 1 h and 3 h, GR protein expression increased to 1.4- and 2.2-fold, respectively, compared to untreated macrophages. These effects were inhibited by RU486. However, mineralocorticoid receptor (MR) protein expression was not influenced by 150 nM corticosterone. These results demonstrate that low concentrations of corticosterone exert stimulatory effects on macrophage function in the absence of immune stimuli, and GR is at least partially responsible for these effects.

## 1. Introduction

Corticosterone is one of the major endogenous glucocorticoids (GCs) secreted from the adrenal cortex in rodents. Due to their potential immune suppressive properties, synthetic GCs have been used in the treatment of a variety of inflammatory and immune diseases. However, accumulating evidence suggests that endogenous GCs, distinct from synthetic GCs, have both stimulatory and suppressive effects on immune cells [1–3]. Although data from *in vitro* studies have revealed that the actual concentration of endogenous GCs may account for these opposing effects [4, 5], endogenous GCs exert stimulatory effects at low concentrations and exert suppressive effects at high concentrations. However, the mechanisms underlying the stimulatory effects of endogenous GCs remain elusive.

The physiological effects of corticosterone are usually mediated by glucocorticoid receptors (GRs) and mineralocorticoid receptors (MRs), both of which are ligand-activated transcription factors. Some studies have shown that MRs play a major role at physiological lower concentrations of corticosterone, whereas GRs are mainly activated at higher concentrations of corticosterone [6–8]. However, other studies have shown that the stimulatory effects on immune cell function by low concentrations of corticosterone are mediated by GRs in the presence of bacterial lipopolysaccharide (LPS) and interferon-*γ* (IFN-*γ*) [4]. MRs and GRs are expressed in many types of immune cells, including macrophages, and they play important roles in the regulation of the immune system by corticosterone. In fact, MRs and GRs expression profiles in macrophages are divergently regulated by immune stimuli. LPS stimulation may induce GR gene expression five-fold above the baseline, whereas MR gene expression is completely inhibited within four hours [9]. Therefore, under moderate physiological stress, particularly in the absence of immune stimuli, the effects and mechanisms of low concentrations of corticosterone on immune cell function may be different from those in the presence of immune stimuli.

 A recent study from our group demonstrated that low concentrations of corticosterone exerted stimulatory effects on macrophage function in the absence of immune stimuli [10]. However, the underlying mechanisms, particularly the contribution of steroid receptors, are poorly understood. In the present study, we aimed to explore the mechanisms underlying the stimulatory effects of corticosterone on macrophage function. Our results showed that the chemotactic and phagocytic activities of rat peritoneal macrophages were highly regulated by corticosterone in a concentration-dependent manner. In particular, macrophage chemotaxis and phagocytosis were markedly enhanced by low concentrations of corticosterone, while the GR antagonist RU486 abolished these effects. Taken together, these data indicate that the stimulatory effects on macrophage function by low concentrations of corticosterone are mainly mediated by GRs.

## 2. Materials and Methods

### 2.1. Animals

Male Sprague-Dawley (SD) rats, 7 to 8 weeks old, were kept individually in hanging wire mesh cages in an accredited animal facility at the Experimental Animal Center of Daping Hospital of the Third Military Medical University (Chongqing, China). The animals were housed in a controlled temperature (22–26°C) and 45–55% humidity. The animal room was maintained on a 12 h light-dark cycle (lights on at 7:00 AM and off at 7:00 PM). All animals were given rat chow and water *ad libitum*. The experimental procedures were approved by the Institutional Animal Care and Use Committee of the Third Military Medical University.

### 2.2. Reagents

Roswell Park Memorial Institute (RPMI) 1640 medium was obtained from Hyclone (Logan, Utah, USA). L-glutamine was obtained from Flow Laboratories (North Ryde, Australia). Polycarbonate membrane with 5 *μ*m pores was purchased from NeuroProbe Inc. (Gaithersburg MD, USA). Formyl-methionyl-leucyl-phenylalanine (FMLP), corticosterone, RU486 (mifepristone), trypan blue, and hematoxylin-eosin were obtained from Sigma (St. Louis, MO, USA). Bovine serum albumin (BSA) was purchased from Roche Diagnostics (Mannheim, Germany). HEPES was obtained from Promega (USA). Antibody against *β*-actin, GR, and MR, and secondary antibodies, goat anti-mouse IgG-HRP and goat anti-rabbit IgG-HRP were purchased from Santa Cruz Biotechnology (Santa Cruz, CA, USA). M-PER Mammalian protein extraction reagent was purchased from Pierce Biotechnology (USA). Polyvinylidene difluoride (PVDF) membranes were obtained from Invitrogen (USA). All chemical reagents were of analytical grade.

### 2.3. Preparation of Peritoneal Macrophages

Rat peritoneal macrophages were obtained via lavage of the peritoneal cavity with ice-cold phosphate-buffered saline (PBS, pH 7.4). All procedures were conducted under aseptic conditions. The cell suspensions were centrifuged at 800 g at 4°C for 5 min. The pellet was washed once with ice-cold PBS and then suspended in RPMI 1640 medium supplemented with 20 mM L-glutamine, 100 U/mL penicillin-streptomycin, and 1% BSA. The number and viability of the peritoneal cells were evaluated visually by the trypan blue staining exclusion method. The cell suspensions were mixed (at the 1 : 1 ratio) with 0.4% trypan blue and then the number of nonviable cells per a total of 100 cells was counted. More than 95% of the cells were macrophages, as judged by morphology staining with Wright-stained smears in all experimental conditions.

### 2.4. Chemotaxis Assay

The chemotaxis assay was performed in a 48-well modified Boyden microchemotaxis chamber using a polyvinylpyrrolidone-free polycarbonate membrane filter with 5 *μ*m pores. To induce chemotaxis, the lower wells were filled with 28 *μ*L RPMI 1640-BSA containing formyl-methionyl-leucyl-phenylalanine (FMLP, 10 nM), which is a bacterial peptide that is a general chemoattractant and attracts various immune cells [11]. The upper wells were filled with 50 *μ*L of the macrophage suspension (3.0 × 10^5^ cells/well) treated with different concentrations of corticosterone. To inhibit the activity of GR, macrophages were preincubated with RU486 (10 *μ*M) at 37°C for 30 min before treatment with corticosterone (150 nM). Macrophages were treated with vehicle diluent (ethanol) as a control group. After incubation in humidified air containing 5% CO_2_ at 37°C for 3 h, the membrane filter was removed and the nonmigrated cells were scraped. The filter was fixed with 4% paraformaldehyde for 20 min, stained with hematoxylin-eosin to define the cell nuclei, and then the cells were mounted on a glass slide. Macrophage chemotaxis was assessed by counting the number of migrated cells with a 40× objective and a 10× ocular. Migration was expressed as the chemotactic index per high-power field (at 400× magnification), which represented the mean number of macrophages in five random microscopy fields per well of the lower face of the filter.

### 2.5. Phagocytosis Assay

For the phagocytosis assay, rat peritoneal macrophages were suspended in RPMI 1640 medium to 1 × 10^6^ cells/mL and seeded in 96-well plates (Costar, Cambridge, MA, USA) at 100 *μ*L/well. The cells were allowed to attach for 2 h at 37°C in a humidified 5% CO_2_ incubator. Then, the cells were washed with PBS three times to remove nonadherent cells. The cells were treated with different concentrations of corticosterone for 1 h. To inhibit the activity of GR, macrophages were preincubated with RU486 (10 *μ*M) at 37°C for 30 min before treatment with corticosterone (150 nM). Macrophages were treated with vehicle diluent (ethanol) as a control group. After washing with PBS, killed *E. coli* BI21 (DE3) suspended in RPMI 1640 medium was added to the peritoneal macrophages at a ratio of 10 bacteria per macrophage. *E. coli* BI21 (DE3), which expresses dsRed-tagged ovalbumin, was generously provided by Dr. Ying Wan (Institute of Immunology, Third Military Medical University, China). After incubation for 30 min at 37°C under 5% CO_2_, the plates were washed with PBS five times and fixed with 4% paraformaldehyde. Phagocytosis of *E. coli *BI21 (DE3) was observed by confocal microscopy (TCS-SP2, Leica Microsystems, Wetzlar, Germany). For each well, macrophages were observed without any predetermined sequence or system. Results were expressed as the phagocytic rate (the percentage of macrophages containing at least one ingested bacterium relative to the total number of macrophages) and the phagocytic index (the mean number of bacteria detected in the cytoplasm of macrophages).

### 2.6. Western Blot Assay

Total protein was extracted from rat peritoneal macrophages by using M-PER mammalian protein extraction reagent, then protein concentration was determined by a Bradford assay. Total protein (50 *μ*g) was loaded per lane and separated by 7.5% sodium dodecyl sulfate-polyacrylamide gel electrophoresis (SDS-PAGE) and then transferred to PVDF membrane. The membrane was blocked with TBS (20 mM Tris-HCl pH 7.6, 150 mM NaCl) containing 5% nonfat dry milk for 2 h at room temperature and then incubated overnight with anti-GR (1 : 500), anti-MR (1 : 500), or anti-*β*-actin (1 : 800) antibodies overnight at 4°C. The membrane was then incubated with a proper secondary antibody and visualized by chemiluminescence. Densitometry analyses were done using Gel-Pro 4.0 image analysis software (Media Cybernetics, Silver Spring, MD). The absorbance ratio of each protein to the reference protein (*β*-actin) was represented as the relative amount of target proteins.

### 2.7. Statistical Analysis

All experiments were performed at least three times, and the results presented were from representative experiments. All data were analyzed by SPSS13.0 statistical package (Chicago, Illinois, USA). The results of parametric data were expressed as means ± standard deviation (SD). Simple pairwise comparisons were performed using Student's *t*-test. Differences were considered statistically significant if the *P* value was <0.05.

## 3. Results

### 3.1. Corticosterone Regulated Macrophage Chemotaxis in a Concentration-Dependent Manner

**Figure 1:**
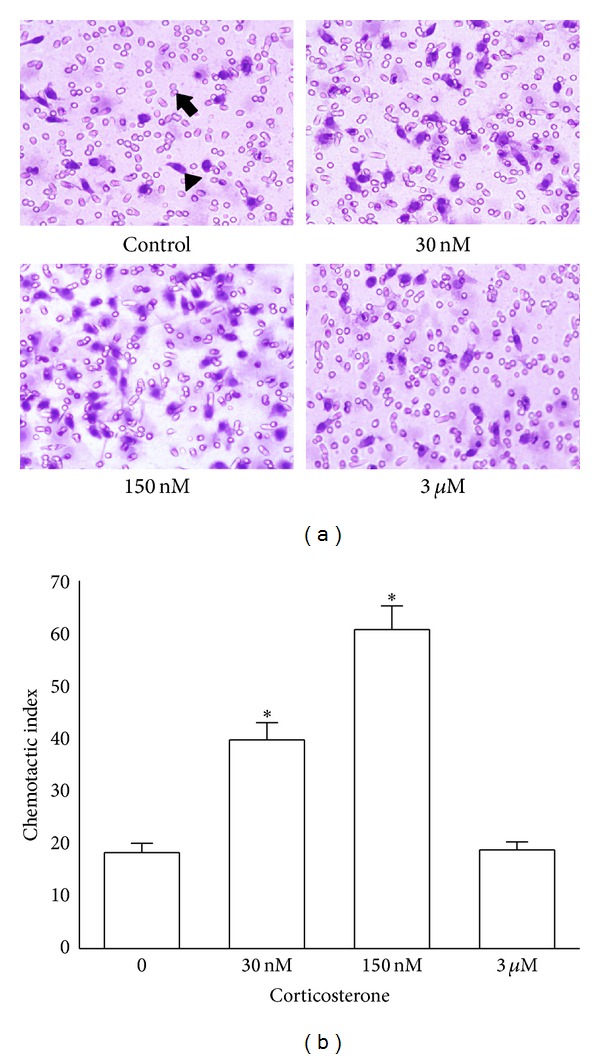
Effects of corticosterone on the chemotaxis of rat peritoneal macrophages. The upper and lower chambers were separated by a 5 *μ*m pore-sized (arrow) polycarbonate membrane filter (polyvinylpyrrolidone-free). FMLP (10 nM) was added into the lower wells to induce cell migration. The filled chamber was incubated at 37°C for 3 h and then the cells that had not migrated into the lower chamber were scraped off. The filter was fixed with 4% paraformaldehyde for 20 min and the cells were stained with hematoxylin-eosin. The migrated cells (arrowhead) were counted under microscopy (×400). (a) Rat peritoneal macrophages were treated with vehicle diluent (ethanol) as control. Macrophages were treated with 30 nM, 150 nM, and 3 *μ*M corticosterone, respectively. (b) The results were representative of five independent experiments performed on triplicate samples. *P < 0.01 versus the control group.

To investigate the effects of different concentrations of corticosterone on the chemotactic activity of macrophages, we chose 30 nM, 150 nM and 3 *μ*M corticosterone to cover a subphysiologically and pathologically relevant range. As shown in Figure 1, rat peritoneal macrophages migrated through 5 *μ*m pores of polyvinylpyrrolidone-free polycarbonate membrane filter in response to FMLP in the absence of immune stimuli. The chemotactic activity of rat peritoneal macrophages was significantly increased after treatment with 30 nM or 150 nM corticosterone. Peritoneal macrophages incubated with low concentrations of corticosterone (30 and 150 nM) showed a greater chemotaxis index than the control group (*P* < 0.01). However, the change in chemotaxis was not observed at a high concentration of corticosterone (3 *μ*M). These results suggest that macrophage chemotaxis is highly regulated by corticosterone in a concentration-dependent manner, and low concentrations of corticosterone could enhance macrophage chemotaxis in the absence of immune stimuli.

### 3.2. Blocking GR Activity Prevented the Enhancement of Macrophage Chemotaxis by Low Concentrations of Corticosterone

**Figure 2:**
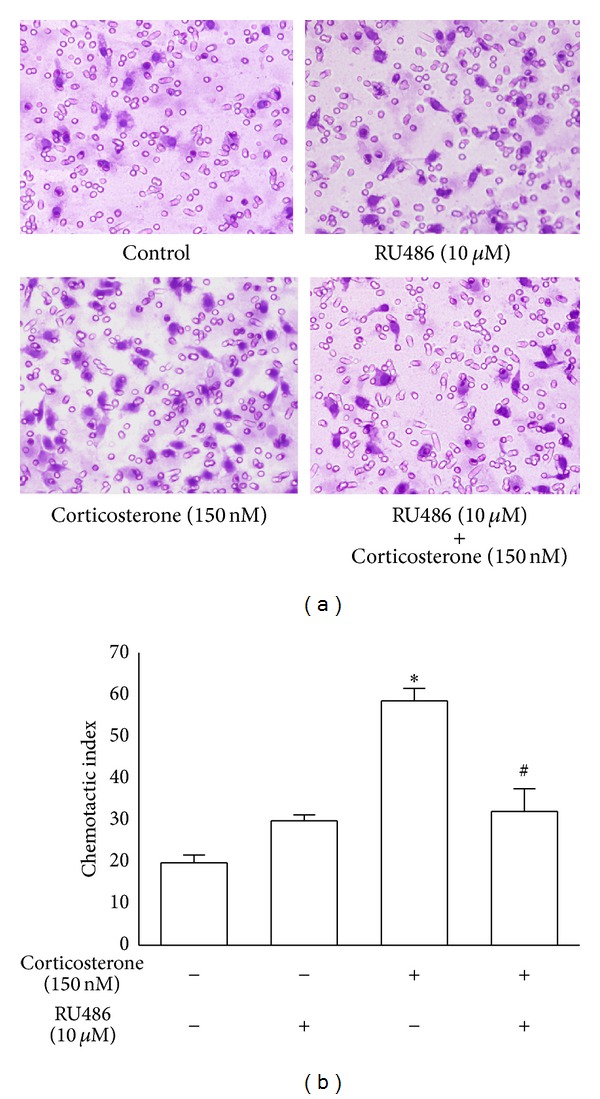
Effects of low concentration of corticosterone on the chemotaxis of rat peritoneal macrophages in the presence or absence of RU486. (a) Rat peritoneal macrophages were treated with vehicle diluent (ethanol) as a control group. Macrophages were treated with RU486 (10 *μ*M), corticosterone (150 nM), and RU486 (10 *μ*M) plus corticosterone (150 nM), respectively (×400). (b) The results were representative of five independent experiments performed on triplicate samples. *P < 0.01 versus the control group, ^#^ *P* < 0.01 versus the corticosterone (150 nM) group.

To better understand the underlying mechanisms of the stimulatory effects of corticosterone, we determined whether GRs were involved in this process. RU486, a potent GR antagonist that inhibits GR-mediated transactivation, was employed to treat peritoneal macrophages. After preincubation with 10 *μ*M RU486, corticosterone (150 nM) could not markedly induce the enhancement of macrophage chemotactic activity, and the chemotaxis index was significantly decreased (*P* < 0.01, Figure 2). These results suggest that GRs may mediate the enhancement of macrophage chemotaxis by low concentrations of corticosterone.

### 3.3. Corticosterone Regulated Macrophage Phagocytosis in a Concentration-Dependent Manner

**Figure 3:**
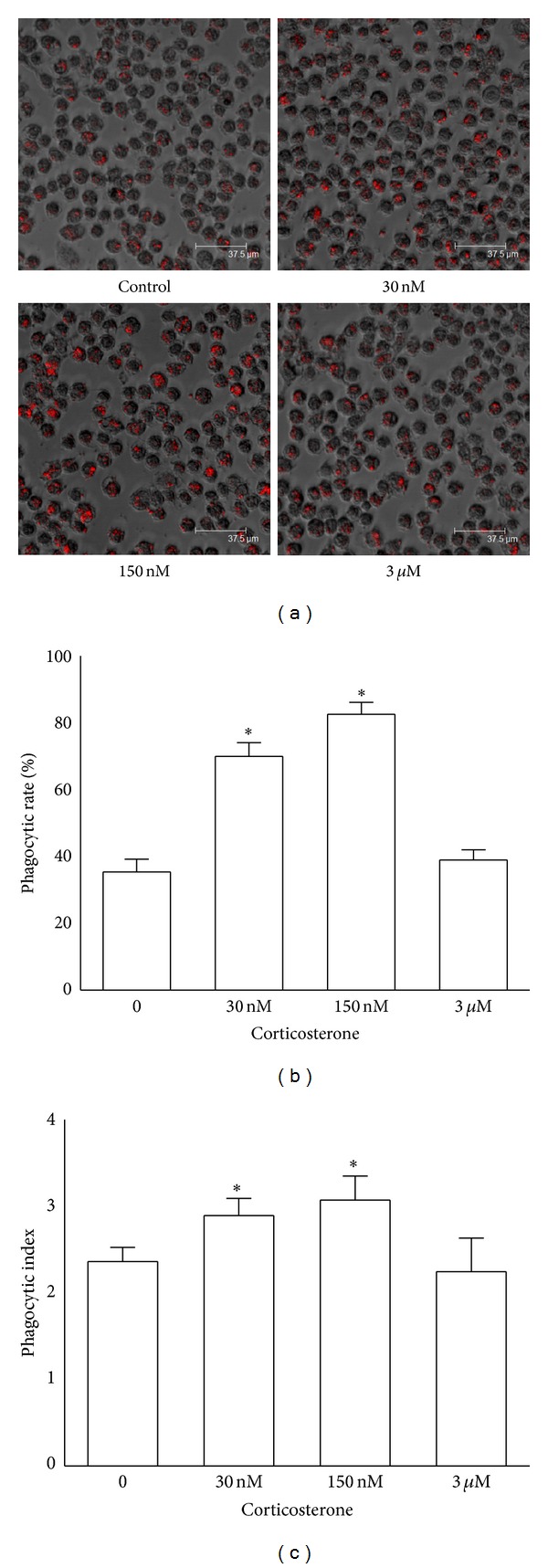
Effects of corticosterone on the phagocytosis of rat peritoneal macrophages. Rat peritoneal macrophages were cultured in 96-well plates and then incubated with bacteria expressing dsRed-tagged ovalbumin for 30 min at 37°C under 5% CO_2_. The macrophages were fixed using 4% paraformaldehyde. The phagocytic activity was determined by evaluating the ability to intake bacteria using confocal microscopy. (a) Rat peritoneal macrophages were treated with vehicle diluent (ethanol) as a control group. Macrophages were treated with 30 nM, 150 nM, and 3 *μ*M corticosterone, respectively. Scale bar, 37.5 *μ*m. The phagocytic activity was estimated by the phagocytic rate (b) and index (c). The results were representative of five independent experiments performed on triplicate samples. *P < 0.01 versus the control group.

Phagocytic activity of rat peritoneal macrophages was determined by incubating cells with bacteria expressing dsRed-tagged ovalbumin. The confocal microscopy assay showed that the phagocytic activity of rat peritoneal macrophages after treatment with 30 nM or 150 nM corticosterone was significantly enhanced when compared to the control group. The phagocytic rate and index of macrophages treated with low concentrations of corticosterone (30 nM or 150 nM) were significantly higher than those of macrophages treated with vehicle diluent (ethanol) (*P* < 0.01, Figure 3). However, statistical differences were not observed between the corticosterone (3 *μ*M) group and the control group (Figure 3). These results indicate that macrophage phagocytosis is highly regulated by corticosterone in a concentration-dependent manner, and low concentrations of corticosterone could enhance macrophage phagocytosis in the absence of immune stimuli.

### 3.4. Blocking GR Activity Prevented the Enhancement of Macrophage Phagocytosis by Low Concentrations of Corticosterone

**Figure 4:**
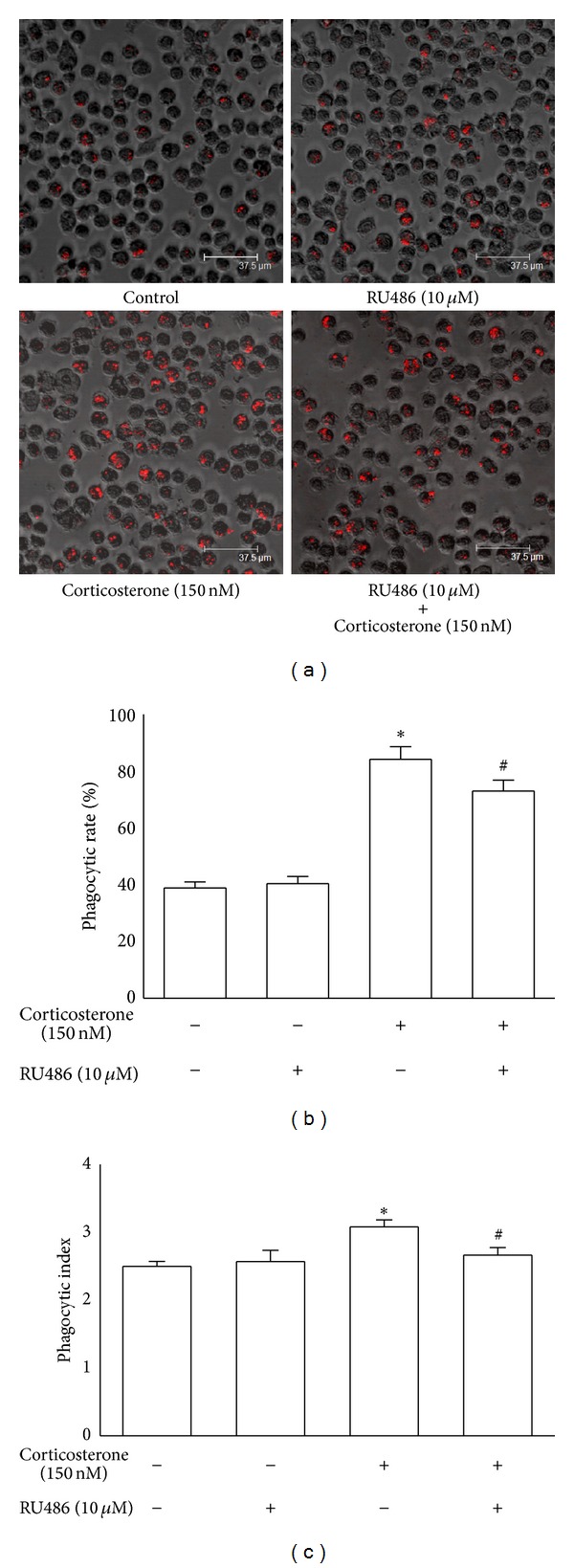
Effects of low concentration of corticosterone on the phagocytosis of rat peritoneal macrophages in the presence or absence of RU486. (a) Rat peritoneal macrophages were treated with vehicle diluent (ethanol) as a control group. Macrophages were treated with RU486 (10 *μ*M), corticosterone (150 nM), and RU486 (10 *μ*M) plus corticosterone (150 nM), respectively. Scale bar, 37.5 *μ*m. The phagocytic activity was estimated by the phagocytic rate (b) and index (c). The results were representative of five independent experiments performed on triplicate samples. *P < 0.01 versus the control group, ^#^ *P* < 0.01 versus the corticosterone (150 nM) group.

To determine whether the enhancement of macrophage phagocytosis by low concentrations of corticosterone is mediated by GRs, rat peritoneal macrophages were preincubated with RU486 (10 *μ*M) for 30 min and then macrophages were treated with corticosterone (150 nM) for 1 h. The phagocytic rate and index were significantly increased in the 150 nM corticosterone group when compared to the control group (*P* < 0.01, Figure 4). However, the phagocytic rate and index were significantly decreased when macrophages were preincubated with 10 *μ*M RU486 (*P* < 0.01, Figure 4). These results suggest that GRs may mediate the enhancement of macrophage phagocytosis by low concentrations of corticosterone.

### 3.5. Low Concentration of Corticosterone Differentially Modulates GR and MR Protein Expression

**Figure 5:**
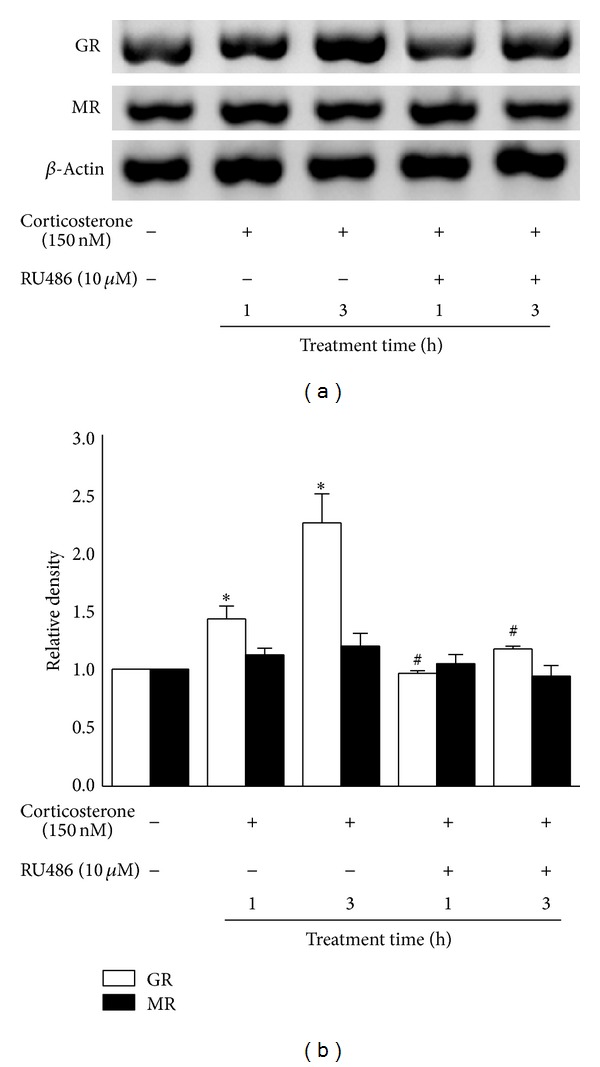
Effects of low concentration of corticosterone on the expression of GR and MR protein in rat peritoneal macrophages. (a) The expression of GR and MR protein was detected by Western blot assay. *β*-actin was used as loading control. (b) Relative levels of GR and MR protein in rat peritoneal macrophages after treatment with corticosterone (150 nM) and RU486 (10 *μ*M). The bars represented the mean ± SD (*n* = 3). *P < 0.05 versus the control group; ^#^ *P* < 0.05 versus macrophages treated with corticosterone (150 nM) for the corresponding time.

We further examined whether GR protein expression could be responsible for low concentration of corticosterone-induced immunostimulatory effects. As show in Figure 5, after treatment with 150 nM corticosterone for 1 h and 3 h, GR protein expression increased to 1.4- and 2.2-fold, respectively, compared to untreated rat peritoneal macrophages. Furthermore, GR antagonist RU486 abolished these effects. However, Western blot assay did not reveal the induction of MR protein expression by 150 nM corticosterone. These results showed that low concentration of corticosterone induced a gradual increase in GR protein expression, but had no effect on MR protein expression in rat peritoneal macrophages.

## 4. Discussion

In this study, we demonstrate that corticosterone, the principal endogenous GC, regulates macrophage function (chemotaxis and phagocytosis) in a concentration-dependent manner in the absence of immune stimuli. Low concentrations of corticosterone significantly enhance macrophage function. Furthermore, blocking GR activity can inhibit these stimulatory effects on macrophages.

Steroid receptors, a superfamily of ligand-activated transcription factors, are gaining increasing attention as important transcription factors in the regulation of immune and inflammatory responses. Androgen receptor activation exerts the anti-inflammatory effect on human benign prostatic hyperplasia cells [12, 13]. Progesterone receptor is confirmed as an anti-inflammatory agent in the endothelium, by downregulating immune cells trafficking into tissues [14]. A number of studies have clearly indicated that GR is involved in GC-mediated anti-inflammatory and immunosuppressive effects [15]. Interestingly, in the present study, our results provide evidence supporting the hypothesis that low concentrations of corticosterone can enhance immune function in the naive macrophages, which may be associated with GR.

 When determining the physiological effects of GCs on immune cells, it is worth noting that these effects mainly depend on the actual concentrations of GCs [4, 16]. In the previous literatures, physiological concentrations of GCs (350 nM to 950 nM) induced the immunoenhancing effects, whereas pharmacological concentrations could inhibit immune cell function [16]. In this study, to examine the concentration-dependent effects of corticosterone on immune cell function, rat peritoneal macrophages were exposed to subphysiological concentrations (30 and 150 nM) and supraphysiological/pathological concentrations (3 *μ*M) of corticosterone in the absence of immune stimuli. The naive macrophages were used to simulate the early phase of stress without immune stimuli. Low concentrations of corticosterone (30 and 150 nM) significantly enhanced the chemotactic and phagocytic activities of macrophages, whereas high concentration (3 *μ*M) did not influence macrophage function in the absence of immune stimuli. Our data are consistent with previous studies showing the concentration-dependent effects of corticosterone on immune cell function [3, 4, 10]. Taken together, these results indicate that natural or endogenous GCs exert physiological effects on immune cells, dependent on their actual concentration.

 Corticosterone exerts its effects mainly through two distinct receptor subtypes, the GRs and MRs, which exhibit different affinities for corticosterone [17]. Many types of steroid receptors are expressed in macrophages, including GRs and MRs [9]. RU486 is a high-affinity antagonist of GRs. A 10-fold excess of RU486 above the GC concentration was sufficient to antagonize the GR effects [18]. In this study, rat peritoneal macrophages were preincubated with 10 *μ*M RU486 for 30 min, which could completely prevent corticosterone from binding to GRs. The final concentration of RU486 was sufficient to antagonize corticosterone-induced GR activation. Our data demonstrated that macrophages preincubated with RU486 could impede the enhancement of macrophage chemotaxis and phagocytosis by low concentration of corticosterone (150 nM), indicating that the stimulatory effects on macrophage function are mediated by GRs in the absence of immune stimuli. A previous study reported that the opposing effects induced by different GC concentrations in peritoneal macrophages were mediated by GR in the presence of LPS and IFN-*γ* stimulation [4]. Therefore, our findings further highlight that GR plays an important role in mediating the stimulatory effects on immune cell function by low concentrations of corticosterone.

In the present study, we examined the effect of low concentration of corticosterone on GR protein expression. Western bolt assay showed that 150 nM corticosterone induced a significant increase in GR protein expression in rat peritoneal macrophages. GR protein expression increased to 1.4-fold or 2.2-fold after treatment with 150 nM corticosterone for 1 h or 3 h, respectively. Preincubation with RU486, an antagonist of GR, abolished these effects. However, MR protein expression was no affected by low concentration of corticosterone. These results are consistent with the previous studies, which have revealed that postnatal handling significantly increase GR gene expression in the rat hippocampus, while handling has no effect on MR gene expression [19]. Some authors believe that the observed increase in GR expression is due to the touch stimulation-induced decrease of corticosterone [20]. Therefore, in the present study, GR may play an important role in low concentration of corticosterone-induced immunostimulatory effects in naive macrophages.

In addition, there is a difference in the effect of RU486 on chemotaxis and phagocytosis of macrophages treated with 150 nM corticosterone. The results of Western blot assay have revealed that GR protein expression increased to 1.4-fold in macrophages treated for 1 h, compared to the control cells, while GR protein expression increased to 2.2-fold in 3 h group. Therefore, this difference may be attributed to the different GR protein expression in rat peritoneal macrophages after treatment for 1 h and 3 h. Furthermore, RU486 is a ligand that acts both as a partial GR agonist and antagonist [21, 22]. Although there is no statistical significance, the results indicated that RU486 can enhance macrophage chemotaxis and phagocytosis (shown in Figures 2 and 4). Because of the higher concentration of RU486, we observed that the immune function of macrophages treated with RU486 plus corticosterone was still enhanced, compared with the control cells. Due to these reasons, the effect of RU486 on chemotaxis was more than the phagocytosis in the present experimental model.

 Nevertheless, it is proposed that MR plays major roles at physiological lower concentrations of corticosterone [7, 8]. The steroid MRs and GRs, which act as ligand-activated transcription factors, play important physiological and pathophysiological roles in a broad range of cell types, including monocytes and macrophages [23]. Due to a 57% homology in the ligand binding domain and 94% in the DNA binding domain between MRs and GRs [24], cross-reactions with corticosterone have been exhibited [23]. Corticosterone can regulate macrophage function through both MRs and GRs. It has been reported that corticosterone exerted effects in a concentration-dependent manner in rat microglial cells, a resident cell of the brain exhibiting similar properties to peripheral macrophages. The stimulatory effects produced by low concentrations of corticosterone are mediated by MRs, whereas the suppressive effects of high concentrations are produced through GRs [25]. These results are inconsistent with our data and a previous study [4]. Actually, GRs are ubiquitously expressed in many cell types, whereas MR expression appears more restricted [26]. 

 Although our data suggest that the stimulatory effects of low concentrations of corticosterone are mediated by GRs, we could not exclude other receptors or molecules that contribute to the enhancement of macrophage function by low concentrations of corticosterone. Recently, emerging evidence indicates that endoplasmic reticulum (ER) stress in immune cells plays an important role in the regulation of cellular functions, such as plasma cell differentiation [27], antigen presentation [28], and IgM synthesis and secretion [29]. Additionally, there is increasing evidence indicating a strong link between ER stress and innate immune response [30, 31]. ER stress-induced transcription factors, such as X-box binding protein 1 (XBP1) and activating transcription factor 6 (ATF6), have been demonstrated to play an essential role in ER stress-induced changes in cellular functions. We speculate that ER stress may play an important role in the immunostimulatory effects in immune cells induced by low concentration of corticosterone. Our further studies will investigate whether the low concentration of corticosterone-induced immunostimulatory effects are mediated by ER stress and the potential molecular mechanisms.

## 5. Conclusion

Taken together, our results show that low concentrations of corticosterone can enhance macrophage chemotaxis and phagocytosis in the absence of immune stimuli, and GR is at least partially responsible for these stimulatory effects. Corticosterone is an important effector hormone for the hypothalamic-pituitary-adrenal axis during stress, and thus it may prime the immune system at the early phase of stress and contribute to enhanced defense against potential challenges.

## References

[1] Yeager MP, Guyre PM, Munck AU (2004). Glucocorticoid regulation of the inflammatory response to injury. *Acta Anaesthesiologica Scandinavica*.

[2] Wiegers GJ, Croiset G, Reul JM, Holsboer F, de Kloet ER (1993). Differential effects of corticosteroids on rat peripheral blood T-lymphocyte mitogenesis in vivo and in vitro. *American Journal of Physiology: Endocrinology and Metabolism*.

[3] Dhabhar FS, McEwen BS (1999). Enhancing versus suppressive effects of stress hormones on skin immune function. *Proceedings of the National Academy of Sciences of the United States of America*.

[4] Lim H-Y, Müller N, Herold MJ, van den Brandt J, Reichardt HM (2007). Glucocorticoids exert opposing effects on macrophage function dependent on their concentration. *Immunology*.

[5] Liao J, Keiser JA, Scales WE, Kunkel SL, Kluger MJ (1995). Role of corticosterone in TNF and IL-6 production in isolated perfused rat liver. *American Journal of Physiology: Regulatory, Integrative and Comparative Physiology*.

[6] Joëls M, de Kloet ER (1994). Mineralocorticoid and glucocorticoid receptors in the brain. Implications for ion permeability and transmitter systems. *Progress in Neurobiology*.

[7] de Kloet ER, Joëls M, Holsboer F (2005). Stress and the brain: from adaptation to disease. *Nature Reviews Neuroscience*.

[8] Nishi M, Kawata M (2007). Dynamics of glucocorticoid receptor and mineralocorticoid receptor: implications from live cell imaging studies. *Neuroendocrinology*.

[9] Barish GD, Downes M, Alaynick WA (2005). A nuclear receptor atlas: macrophage activation. *Molecular Endocrinology*.

[10] Zhou J-Y, Zhong H-J, Yang C, Yan J, Wang H-Y, Jiang J-X (2010). Corticosterone exerts immunostimulatory effects on macrophages via endoplasmic reticulum stress. *British Journal of Surgery*.

[11] Ortega E, Forner MA, Barriga C (1997). Exercise-induced stimulation of murine macrophage chemotaxis: role of corticosterone and prolactin as mediators. *Journal of Physiology*.

[12] Vignozzi L, Cellai I, Santi R (2012). Antiinflammatory effect of androgen receptor activation in human benign prostatic hyperplasia cells. *The Journal of Endocrinology*.

[13] Vignozzi L, Gacci M, Cellai I (2013). Fat boosts, while androgen receptor activation counteracts, BPH-associated prostate inflammation. *The Prostate*.

[14] Goddard LM, Ton AN, Org T, Mikkola HK, Iruela-Arispe ML (2013). Selective suppression of endothelial cytokine production by progesterone receptor. *Vascular Pharmacology*.

[15] Tuckermann JP, Kleiman A, McPherson KG, Reichardt HM (2005). Molecular mechanisms of glucocorticoids in the control of inflammation and lymphocyte apoptosis. *Critical Reviews in Clinical Laboratory Sciences*.

[16] Webster JI, Tonelli L, Sternberg EM (2002). Neuroendocrine regulation of immunity. *Annual Review of Immunology*.

[17] Joëls M, de Kloet ER (1992). Coordinative mineralocorticoid and glucocorticoid receptor-mediated control of responses to serotonin in rat hippocampus. *Neuroendocrinology*.

[18] Honer C, Nam K, Fink C (2003). Glucocorticoid receptor antagonism by cyproterone acetate and RU486. *Molecular Pharmacology*.

[19] O’Donnell D, Larocque S, Seckl JR, Meaney MJ (1994). Postnatal handling alters glucocorticoid, but not mineralocorticoid messenger RNA expression in the hippocampus of adult rats. *Molecular Brain Research*.

[20] Jutapakdeegul N, Casalotti SO, Govitrapong P, Kotchabhakdi N (2003). Postnatal touch stimulation acutely alters corticosterone levels and glucocorticoid receptor gene expression in the neonatal rat. *Developmental Neuroscience*.

[21] Groyer A, Schweizer-Groyer G, Cadepond F, Mariller M, Baulieu EE (1987). Antiglucocorticosteroid effects suggest why steroid hormone is required for receptors to bind DNA in vivo but not in vitro. *Nature*.

[22] Schulz M, Eggert M, Baniahmad A, Dostert A, Heinzel T, Renkawitz R (2002). RU486-induced glucocorticoid receptor agonism is controlled by the receptor N terminus and by corepressor binding. *Journal of Biological Chemistry*.

[23] Rickard AJ, Young MJ (2009). Corticosteroid receptors, macrophages and cardiovascular disease. *Journal of Molecular Endocrinology*.

[24] Arriza JL, Weinberger C, Cerelli G (1987). Cloning of human mineralocorticoid receptor complementary DNA: structural and functional kinship with the glucocorticoid receptor. *Science*.

[25] Tanaka J, Fujita H, Matsuda S, Toku K, Sakanaka M, Maeda N (1997). Glucocorticoid- and mineralocorticoid receptors in microglial cells: the two receptors mediate differential effects of corticosteroids. *Glia*.

[26] Funder JW (2005). Mineralocorticoid receptors: distribution and activation. *Heart Failure Reviews*.

[27] Reimold AM, Iwakoshi NN, Manis J (2001). Plasma cell differentiation requires the transcription factor XBP-1. *Nature*.

[28] de Almeida SF, Fleming JV, Azevedo JE, Carmo-Fonseca M, de Sousa M (2007). Stimulation of an unfolded protein response impairs MHC class I expression. *Journal of Immunology*.

[29] Tirosh B, Iwakoshi NN, Glimcher LH, Ploegh HL (2005). XBP-1 specifically promotes IgM synthesis and secretion, but is dispensable for degradation of glycoproteins in primary B cells. *Journal of Experimental Medicine*.

[30] Martinon F, Glimcher LH (2011). Regulation of innate immunity by signaling pathways emerging from the endoplasmic reticulum. *Current Opinion in Immunology*.

[31] Martinon F, Chen X, Lee A-H, Glimcher LH (2010). TLR activation of the transcription factor XBP1 regulates innate immune responses in macrophages. *Nature Immunology*.

