# Correction to “Low Concentrations of Corticosterone Exert Stimulatory Effects on Macrophage Function in a Manner Dependent on Glucocorticoid Receptors”

**DOI:** 10.1155/ije/9874547

**Published:** 2026-01-30

H.‐J. Zhong, H.‐Y. Wang, C. Yang, J.‐Y. Zhou, and J.‐X. Jiang, “Low Concentrations of Corticosterone Exert Stimulatory Effects on Macrophage Function in a Manner Dependent on Glucocorticoid Receptors,” *International Journal of Endocrinology* 2013, no. 1 (2013): 1–9, https://doi.org/10.1155/2013/405127.

In the article titled “Low Concentrations of Corticosterone Exert Stimulatory Effects on Macrophage Function in a Manner Dependent on Glucocorticoid Receptors,” there was an error in Figure 1(a). More specifically, an incorrect image was selected to depict the macrophages treated with 30 nM corticosterone, which resulted in accidental overlap with the panel showing macrophages treated with RU486 + corticosterone in Figure 2a.

This error has occurred during figure assembly and Figure 1 should be corrected as follows:

Figure 1Effects of corticosterone on the chemotaxis of rat peritoneal macrophages. The upper and lower chambers were separated by a 5‐μm pore‐sized (arrow) polycarbonate membrane filter (polyvinylpyrrolidone‐free). FMLP (10 nM) was added into the lower wells to induce cell migration. The filled chamber was incubated at 37°C for 3 h and then the cells that had not migrated into the lower chamber were scraped off. The filter was fixed with 4% paraformaldehyde for 20 min and the cells were stained with hematoxylin‐eosin. The migrated cells (arrowhead) were counted under microscopy (× 400). (a) Rat peritoneal macrophages were treated with vehicle diluent (ethanol) as control. Macrophages were treated with 30 nM, 150 nM, and 3 μM corticosterone, respectively. (b) The results were representative of five independent experiments performed on triplicate samples. ^∗^ *P*  <  0.01 versus the control group.

**Figure:**
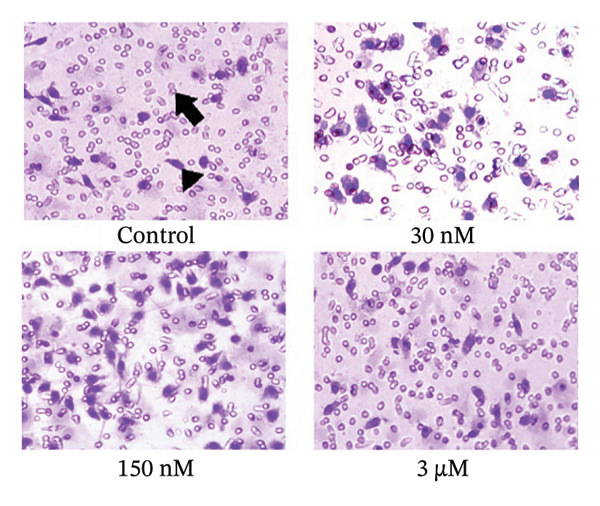
(a)

**Figure:**
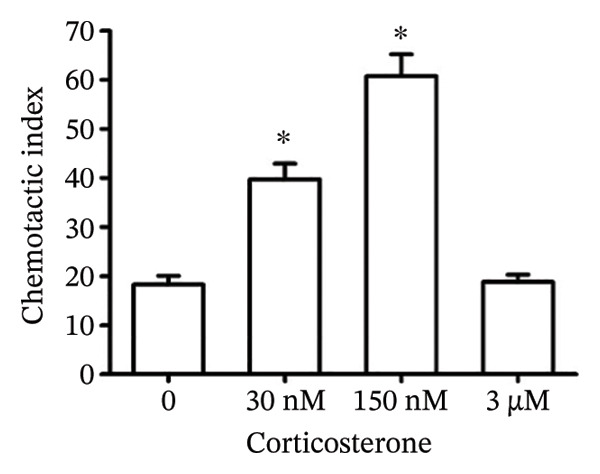
(b)

We apologize for this error.

